# Tumor necrosis factor α in aGVHD patients contributed to the impairment of recipient bone marrow MSC stemness and deficiency of their hematopoiesis-promotion capacity

**DOI:** 10.1186/s13287-020-01615-9

**Published:** 2020-03-17

**Authors:** Li Ding, Hong-Mei Ning, Pei-Lin Li, Hong-Min Yan, Dong-Mei Han, Xiao-Li Zheng, Jing Liu, Ling Zhu, Mei Xue, Ning Mao, Zi-Kuan Guo, Heng Zhu, Heng-Xiang Wang

**Affiliations:** 1 Medical Center of Air Forces, PLA, Road Fucheng 30, Beijing, 100142 People’s Republic of China; 2 Beijing Institute of Radiation Medicine, Road Taiping 27, Beijing, 100850 People’s Republic of China; 3 The Fifth Medical Center of Chinese PLA General Hospital, East Street 8, Beijing, 100071 People’s Republic of China; 4 Beijing Institute of Basic Medical Sciences, Road Taiping 27, Beijing, 100850 People’s Republic of China

**Keywords:** Acute graft versus host disease, Bone marrow niche, Mesenchymal stem cell, Stemness, Tumor necrosis factor-α

## Abstract

**Background:**

Though accumulated evidence has demonstrated visceral organ involvement in acute graft-versus-host disease (aGVHD), how aGVHD influences the bone marrow (BM) niche and the reconstitution of hematopoiesis post-hematopoietic stem cell transplantation remains largely unknown.

**Methods:**

In the current study, the cell morphology, immunophenotype, multi-differentiation capacity, self-renewal capacity, and hematopoiesis promotion of the MSCs from aGVHD and non-aGVHD patients were investigated. Additionally, the stemness and hematopoiesis-promoting property of healthy donor-derived MSCs were evaluated in the presence of BM supernatant from aGVHD patients. Mechanistically, antibodies targeting inflammatory cytokines involved in aGVHD were added into the MSC culture. Furthermore, a recombinant human tumor necrosis factor (TNF-α) receptor-Ig fusion protein (rhTNFR:Fc) was used to protect healthy donor-derived MSCs. Moreover, mRNA sequencing was performed to explore the underlying mechanisms.

**Results:**

The aGVHD MSCs exhibited morphological and immunophenotypic characteristics that were similar to those of the non-aGVHD MSCs. However, the osteogenic and adipogenic activities of the aGVHD MSCs significantly decreased. Additionally, the colony formation capacity and the expression of self-renewal-related genes remarkably decreased in aGVHD MSCs. Further, the hematopoiesis-supporting capacity of aGVHD MSCs significantly reduced. The antibody neutralization results showed that TNF-α contributed to the impairment of MSC properties. Moreover, rhTNFR:Fc exhibited notable protective effects on MSCs in the aGVHD BM supernatants. The mRNA sequencing results indicated that the TNF-α pathway and the Toll-like receptor pathway may be activated by TNF-α.

**Conclusions:**

Thus, our data demonstrate MSCs as cellular targets of aGVHD and suggest a potential role of TNF-α blockage in maintaining the BM niche of aGVHD patients.

## Introduction

For many years, allogeneic stem cell transplantation (allo-HSCT) has been used for the treatment of hematological malignancies and nonmalignant hematologic disorders [1, 2]. However, the success of allo-HSCT is often limited by the development of acute graft-versus-host disease (aGVHD). aGVHD is classically defined by Billingham as a syndrome in which donor immunocompetent cells recognize and attack host tissues in immunocompromised allogeneic recipients [3, 4]. The cytotoxic T cells and damaged tissues released a burst of cytokine storm, including inflammatory cytokines such as tumor necrosis factor alpha (TNF-α), interferon gamma (IFN-γ), and numerous interleukins, which recruit more effector cells that further augment tissue injury and result in a self-perpetuating state of aGVHD [3, 4].

Though the skin, liver, and intestine are regarded as the principal target organs of life-threatening aGVHD, increasing evidence has demonstrated that the bone marrow (BM) is a potential target tissue of aGVHD, which may contribute to long-term cytopenic conditions, immunodeficiency, bleeding, and infections in patients undergoing post-allo-HSCT [5–8]. Numerous studies have demonstrated that aGVHD affects the restoration of almost all hematopoietic lineages post-allo-HSCT, including lymphopoiesis, myelopoiesis, and megakarypoiesis, by targeting HSCs. In addition, accumulated evidence indicates that aGVHD may damage the structure of the stromal niche in BM. Shono et al. reported that the early destruction of osteoblasts especially affected B cell lineages and caused defective B lymphopoiesis [5]. Yao et al. provided evidence that the vascular niche was a target of aGVHD in a major histocompatibility complex (MHC)–haploidentical matched murine HSCT model [9]. However, little information is available about the changes of mesenchymal stem cells (MSCs), which are a major cell origin of BM stromal cells, including osteoblasts and endothelial cells, in clinical cases.

MSCs and their progeny are important supporting cells in the BM niche [10–15]. In our previous study, we reported that recipient MSCs exhibit normal multiple differentiation and hematopoietic supporting functions, which enable them to contribute to BM reconstitution. In addition, we showed the restorative kinetics of MSCs in leukemia patients post-allo-HSCT over 2 years. Most importantly, our data demonstrated that it was recipient-derived MSCs instead of donor MSCs in infused BM that were involved in rebuilding of the BM niche [16]. However, to our best knowledge, the available information about MSC stemness, including self-renewal and multi-differentiation, in aGVHD patients remains controversial. Additionally, little research has been done to investigate the hematopoiesis-promoting capacity of BM MSCs in aGVHD patients. Third, in human cases, it remains unknown which factors cause the change of recipient MSCs and how to decrease their harmful effects to promote rebuilding of the BM niche post-allo-HSCT. Thus, we generated the hypothesis that aGVHD may indirectly hamper the reconstitution of the BM niche and delay hematopoiesis recovery post-allo-HSCT by injuring recipient BM MSCs. In the current study, we tested this hypothesis by comparing the stemness and hematopoiesis-supporting capacity of MSCs from aGVHD patients and non-aGVHD patients. The role of TNF-α in MSC damage was also investigated.

## Materials and methods

### Patients

Thirty patients transplanted with peripheral blood stem cells (PBSCs) and BM cells from allogeneic donors in the General Hospital of the Air Force between February 2010 and October 2013 were enrolled in this study. The median age was 15.5 years (range 4–43 years). Oral consents for publication have been obtained from the patients and patient characteristics are shown in Table S1. This study was approved by the ethics and technological committees of the General Hospital of the Air Force (2010-0182). The bone marrow samples were routinely collected post-HSCT to monitor stem cell chimerism, leukemic relapse, and hematopoietic activities in BM. The redundant samples were used for MSC culture, and informed consent was obtained from all patients and their next of kin. The preparation and infusion of BM and PBSCs were performed as previously reported [16]. Briefly, all donors received human granulocyte colony-stimulating factor (G-CSF) at a dose of 5–10 μ g/kg daily for 5 consecutive days prior to BM collection, and the mean volume of the harvested BM for HSCT was 10 mL/kg. One day post-BM collection, PBSCs were harvested using a blood cell separation instrument (CS-3000 plus) with a total blood volume of 10 L. Nonmanipulated BM and PBSCs were infused separately into the recipient on the day of collection.

### Culture and expansion of aGVHD MSCs

The diagnosis of aGVHD was performed by a scoring system incorporating clinical features including weight loss, activity, skin integrity, and lab examinations [3, 17]. Twelve of fifteen aGVHD patients suffered from intestinal aGVHD and three others showed liver injury. No patients received therapies that targeted TNF-α at the moment of bone marrow collection and MSC isolation in the current study. Human BM samples were aspirated from the iliac crest of aGVHD patients and non-aGVHD patients post-allogeneic HSCT. The BM supernatants were harvested by centrifugation at 400*g* for 10 min and were filtered (0.4 μm) to deplete cellular components. Then, the cell-free samples were stored at − 80 °C until use. The BM mononuclear cells were isolated by gradient centrifugation at 900*g* for 20 min on Percoll (1.073 g/mL, Amersham Biosciences, Uppsala, Sweden). The mononuclear cells were cultured at a density of 2 × 10^5^ cells/cm^2^ with low-glucose Dulbecco’s modified Eagle’s medium (LGDMEM; Invitrogen, Carlsbad, CA) supplemented with 10% fetal bovine serum (FBS; HyClone, Logan, UT). The non-adherent cells were removed by a complete culture media change at 72 h of initial culture. The adherent cells were observed, and the images were captured by a light microscope (Nikon TE2000-U). The cells were trypsinized and harvested (0.05% trypsin at 37 °C for 5 min) when they were confluent at approximately 80%. The cells were reseeded at a split ratio of 1:3. MSCs at passages 3–6 were used for experiments unless otherwise described [16].

### Flow cytometry analysis of aGVHD MSCs

Fluorescein isothiocyanate (FITC)- or phycoerythrin (PE)-conjugated monoclonal antibodies against human CD31, CD44, CD45, CD73, CD105, and CD166 (all products from eBioscience, San Diego) were used to determine the MSC immunophenotype according to previously published protocols [10, 16]. In brief, the MSCs were harvested by trypsin digestion and washed 2 times with PBS before incubation with or without antibodies for 20 min at 4 °C in the dark. Then, each aliquot was washed twice with PBS, and events were acquired by a FACSCalibur instrument (Becton, Dickinson and Company, Franklin Lakes, NJ, http://www.bd.com). The data were analyzed with WinMDI 2.9 software (Joseph Trotter, The Scripps Institute, La Jolla, CA).

### Cell proliferation of aGVHD MSC

The cell proliferation of aGVHD MSCs and non-aGVHD MSCs was evaluated by Cell Counting Kit 8 (CCK-8; Dojindo) and growth kinetics. For the CCK-8 test, MSCs were seeded in 96-well plates (2 × 10^3^/well, five wells in each group) and maintained in LG-DMEM medium with 10% FBS. CCK-8 solution was added at a ratio of 100 μl/ml, and the MSC culture plates were incubated at 37 °C for 1 h. Absorbance was measured at a wavelength of 450 nm by using a microplate reader. In the present study, the CCK-8 assays were performed at different time points at days 1, 3, 5, 7, 9, and 11.

The growth kinetics of MSCs were evaluated by using the trypan blue exclusion cell count method. In brief, all MSCs were cultured in 48-well plates at a cell density of 2 × 10^4^/well (five wells in each groups) and harvested every other day over a period of 12 days for hemocytometer cell counting.

### aGVHD MSC pluripotency differentiation assay

The osteogenic and adipogenic differentiation of aGVHD MSCs and non-aGVHD MSCs were determined by induction agents as previously described [10, 16]. For osteogenic differentiation, MSCs were seeded in 48-well plates at a cell density of 2 × 10^3^/well (five wells in each groups) and incubated in osteogenic induction medium (10 mM glycerol-2-phosphate, 0.1 mM dexamethasone, and 20 mM ascorbic acid) for 14 or 28 days. For adipogenic differentiation, MSCs were seeded in 48-well plates at a cell density of 1 × 10^4^/well (five wells in each groups) and incubated in adipogenic induction medium for 14 days (1 mM isobutylmethylxanthine and 10^–3^ mM dexamethasone). To evaluate osteogenesis, a histochemical kit (Sigma) was used to assess the expression of the osteogenic marker alkaline phosphatase (ALP) according to the manufacturer’s protocol at day 14, and the mineralization activity was evaluated using von Kossa staining at day 28. To evaluate adipogenesis, Oil-Red-O staining was performed at day 14 according to previously described methods. To further determine the multiple differentiation capacity of MSCs, the mRNA expression level of the osteogenic genes Runx-2 and Osteorix and the adipogenic genes CEBP/α and PPARγ in MSCs were determined at day 7 by using quantitative PCR. To investigate the mechanisms of aGVHD on MSC multiple differentiation capacity, the aGVHD BM supernatant (20%, vol/vol), neutralization antibodies (100 μg/ml) against inflammatory cytokines, and a clinically relevant recombinant human tumor necrosis factor (TNF-α) receptor-Ig fusion protein (rhTNFR:Fc) (commercial name etanercept, 100 μg/ml) were added into the healthy donor-derived BM MSC differentiation culture system in some experiments.

### aGVHD MSC self-renewal assay

In the current study, MSC self-renewal was assessed by a colony-forming unit fibroblast formation assay (CFU-F assay) and sphere formation assay. For the primary CFU-F assay, mononuclear cells were prepared by gradient centrifugation from the BM of aGVHD patients (*n* = 6) and non-GVHD patients (*n* = 6). Aliquots (5 × 10^6^/well) of cell suspensions were added in triplicate into six-well culture plates and were maintained in culture for 14 days. Visible colonies larger than 3 mm in diameter were counted under an inverted microscope after crystal violet staining. For the primary sphere formation assay, aliquots (5 × 10^4^/well) of passage 1 MSC suspensions were added in triplicate into ultralow attachment culture dishes (10 cm ^2^ culture area/dish, Corning) and maintained in media for 7 days. Cell spheres larger than 3 mm in diameter were counted under an inverted microscope. For secondary colony formation assays, primary cell colonies in CUF-F and sphere formation experiments were digested and re-seeded these cells to develop cell colonies. Briefly, aliquots (5 × 10^3^/well) of cell suspensions were added in triplicate into six-well culture plates, and CFU-F numbers were determined by crystal violet staining after 14 days. For secondary sphere formation experiments, aliquots (5 × 10^4^/well) of MSC that from primary spheres were added in triplicate into ultralow attachment culture dishes (10 cm^2^ culture area/dish, Corning) and the spheres were counted at 7 days.

In some experiments, the aGVHD BM supernatant (20%, vol/vol), neutralization antibodies (100 μg/ml) against inflammatory cytokines, and a rhTNFR:Fc (100 μg/ml) were added into the healthy donor-derived BM MSC self-renewal culture system to investigate the mechanisms of aGVHD on MSCs.

### Long-term culture of hematopoietic cells on aGVHD MSC feeders

A hematopoietic colony-forming cell (CFC) assay was carried out according to previously published protocols [16]. In brief, aGVHD MSCs and non-aGVHD MSCs were seeded onto 12-well cell culture plates (1 × 10^5^/well) and irradiated (10 Gy) to develop cell feeder layers. CD34-positive hematopoietic progenitor cells were isolated from healthy donor BMs using a CD34-positive cell isolation kit (Miltenyi Biotec, Bergisch Gladbach, Germany) and were seeded at a cell density of 3 × 10^4^/well on MSC feeders and cocultured at 37 °C with 5% CO_2_ for 7 weeks. The CD34-positive hematopoietic progenitor cells cultured without a feeder layer served as a negative control. The culture medium was half-volume changed weekly, and the non-adherent fraction was reseeded in a methylcellulose-based CFC assay system (Stemcell Technologies, Vancouver, Canada). The CFC numbers were counted under light microscope.

Alternatively, the bulk culture of long-term culture-initiating cells (LTC-ICs) was examined by quantifying the CFC according to the manufacturer’s instructions (StemCell Technologies) at the end of 7 weeks of incubation. Briefly, the CD34-positive hematopoietic progenitor cells were cultured on the cell feeder layers that derived from aGVHD MSCs and non-aGVHD MSCs for 5 weeks, respectively. Consequently, the expanded progenitors were harvested and seeded into the methylcellulose-based CFC assay system for another 2 weeks. The CFC formations were observed at the end of 7 weeks. The aGVHD BM supernatant (20%, vol/vol), neutralization antibodies (100 μg/ml) against inflammatory cytokines, and a rhTNFR:Fc (100 μg/ml) were added into the healthy MSC-mediated long-term culture of hematopoietic cells for mechanistic exploration.

### Quantitative PCR

To assess the mRNA expression of self-renewal-related genes in aGVHD MSCs and non-GVHD MSCs, the cells were seeded into 6-well plates at a cell density of 1 × 10^6^/well and starved in serum-free LG-DMEM medium for at least 6 h. The total RNA was extracted with TRIZOL reagent (Invitrogen) and reverse transcribed using the mRNA Selective PCR kit (TaKaRa). Human Nanog, Oct-4, and Sox2 cDNA were amplified by real-time PCR using the SYBR Green PCR kit (Sigma). Meanwhile, the mRNA expression of MSC multiple differentiation-related genes, including Runx-2, Osteorix, CEBP/α, and PPARγ, was determined by real-time PCR in a similar manner. The primer sequences used for the real-time PCR are shown in Table S2.

### Western blotting

aGVHD MSCs and non-aGVHD MSCs were seeded into 6-well plates at a cell density of 1 × 10^6^/well and starved in serum-free LG-DMEM medium for at least 6 h. The medium were removed, and lysis buffer (BioRad, Hercules, CA) was added into the wells of plates. MSC proteins were collected by cell lysis on ice, and thawed lysates were vortexed and centrifuged. Protein concentrations of lysates were determined by using the BCA Protein Assay (Pierce). Proteins were separated by 10% sodium dodecyl sulfate–polyacrylamide gel electrophoresis and transferred onto nitrocellulose membranes. The membranes were blocked by incubation with 5% wt/vol nonfat dry milk. Membranes were incubated with anti-human Nanog, Oct-4, Sox2 (Cell Signaling, Beverly, MA), and β-actin (Sigma) at the appropriate dilution overnight at 4 °C. Horseradish peroxidase-conjugated secondary antibodies were added to the membranes in 5% nonfat dry milk in TBST. Membranes were developed by using an enhanced chemiluminescence kit (Pierce, Rockford, IL).

### RNA sequencing analyses of MSCs

To further investigate the underlying mechanism of TNF-α mediated impairment on MSC properties, the healthy donor-derived MSCs with or without TNF-α incubations (50 ng/ml, 24 h) were collected and performed high-throughput RNA sequencing analyses. Next-generation sequencing library preparations and Illumina MiSeq sequencing were conducted at GENEWIZ, Inc. (Suzhou, China). DNA libraries were validated by Agilent 2100 Bioanalyzer (Agilent Technologies, Palo Alto, CA, USA) and quantified with Qubit 2.0 Fluorometer. DNA libraries were multiplexed and loaded on an Illumina MiSeq instrument according to manufacturer’s instructions (Illumina, San Diego, CA, USA). Sequencing was performed using a 2 × 300 paired-end (PE) configuration; image analysis and base calling were conducted by using the MiSeq Control Software (MCS) embedded in the MiSeq instrument. All differential abundant mRNAs were used for GO analysis to deepen the understanding of molecular mechanism of cell biological information processes.

### ELISA assay for human TNF-α in BM supernatants

Concentration of human TNF-α in BM supernatants of non-aGVHD patients (*n* = 15) and aGVHD patients (*n* = 15) were determined according to the reagent protocols of the quantitative determination kit of human TNF-α (R&D Systems, Minneapolis). Optical density was read at 450 nm. For each experimental culture well, duplicate ELISA readings were obtained.

### Statistical analysis

Data are represented as the mean values with standard deviations. Statistical significance was analyzed using Student’s *t* test and one-way ANOVA. *p* values less than 0.05 were considered to be significant.

## Results

### aGVHD MSCs share similar morphological and immunophenotypic characteristics with non-aGVHD MSCs

In the current study, fibroblast-like MSCs were cultured from all BM samples. Attached cells appeared at day 7, and colonies developed at approximately day 14 (Fig. S1A). The cell layers usually reached 70–80% confluence between 3 and 4 weeks of initial culture. No remarkable difference in cell morphology was observed between aGVHD MSCs and non-aGVHD MSCs.

The immunophenotypes of aGVHD MSCs and non-aGVHD MSCs were determined by flow cytometry. These two kinds of MSCs were negative for an endothelial marker (CD31) and an hematopoietic marker (CD45) but positive for a stromal marker (CD44) and stem cell markers (CD73, CD105, CD166) (Fig. S1B). The data are consistent with the previously reported cell surface marker profile of human BM MSCs.

### aGVHD MSCs exhibited decreased cell proliferation and self-renewal capacity

To investigate whether aGVHD impairs the growth of MSCs, cell proliferation assays were performed in the current study. Trypan blue exclusion cell counting (Fig. S2A) and a CCK-8-based cell proliferation assay (Fig. S2B) showed that aGVHD MSCs exhibited decreased proliferative capacity relative to that of non-aGVHD MSCs (*p < 0.05, **p < 0.01).

**Fig. 1:**
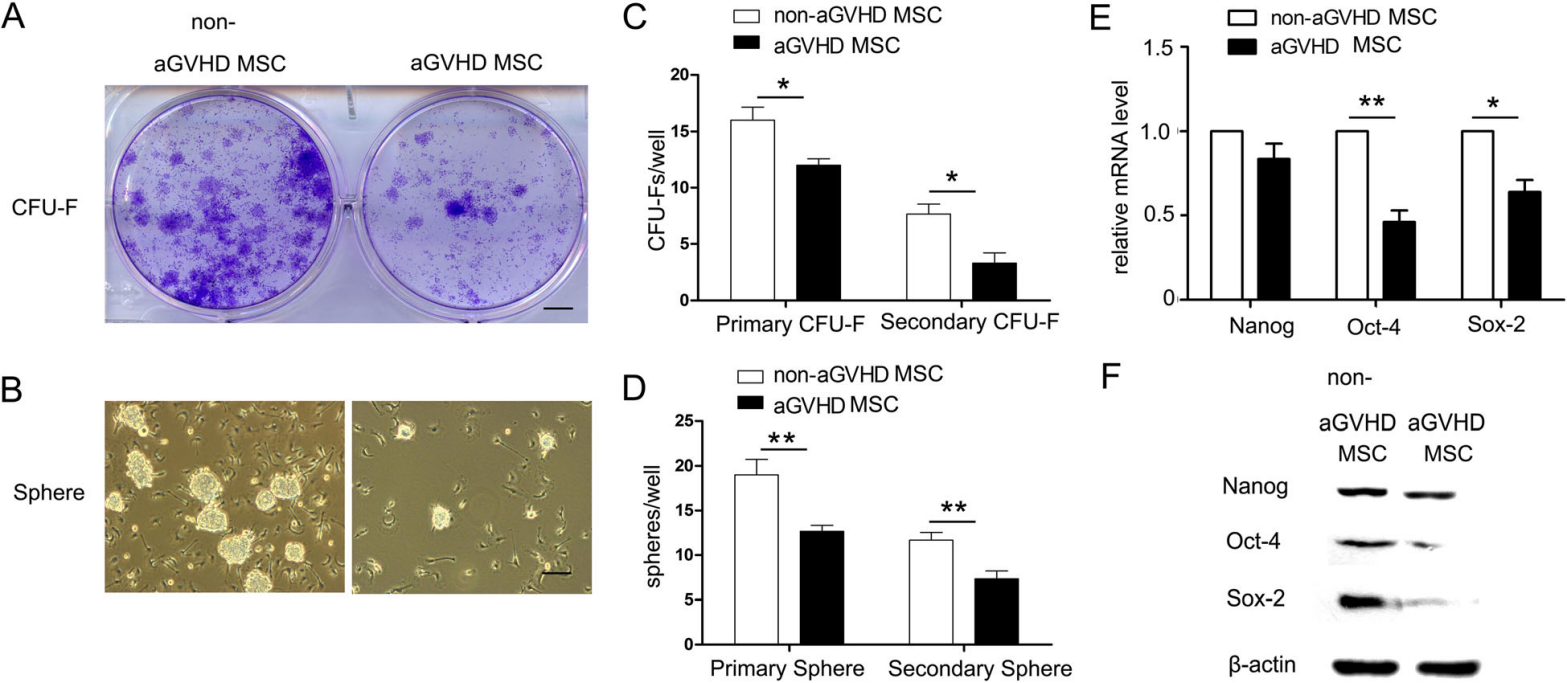
aGVHD MSCs showed impaired self-renewal capacity. **a** Serial CUF-F experiments and **b** secondary sphere formation experiments were performed. Bar in Fig. 3a upper row represents 5 mm. Bar in **a** lower row represent 200 μm. **c** The number of CFU-Fs and **d** cell spheres in aGVHD group was remarkably decreased relative to their counterparts in non-aGVHD group. *p < 0.05, **p < 0.01, *n* = 5. **e** mRNA expression and **f** protein level of Oct-4 and Sox-2 were lower in aGVHD MSCs. *p < 0.05, **p < 0.01, compared with that of non-aGVHD MSCs

In addition, the results of the CFU-F experiments demonstrated that the number of CFU-Fs in aGVHD BM cell culture was remarkably decreased relative to their counterparts in non-aGVHD cell culture (Fig. 1a, c). Furthermore, the data from sphere formation experiments showed that aGVHD caused a remarkable decrease in BM MSC colonies (Fig. 1b, d). Of note, we performed the secondary colony formation experiments by digesting primary cell colonies in CUF-F and sphere formation experiments and re-seeded these cells to develop cell colonies. The results showed that the number of secondary CFU-Fs and spheres reduced in the aGVHD groups, which indicated decreased self-renewal of aGVHD MSCs (Fig. 1c, d). Moreover, the mRNA and protein expression levels of self-renewal-dependent genes, including Oct4 and Sox2, significantly dropped (Fig. 1e, f) (*p < 0.05, **p < 0.01).

### aGVHD MSCs showed impaired osteogenic and adipogenic differentiation

**Fig. 2:**
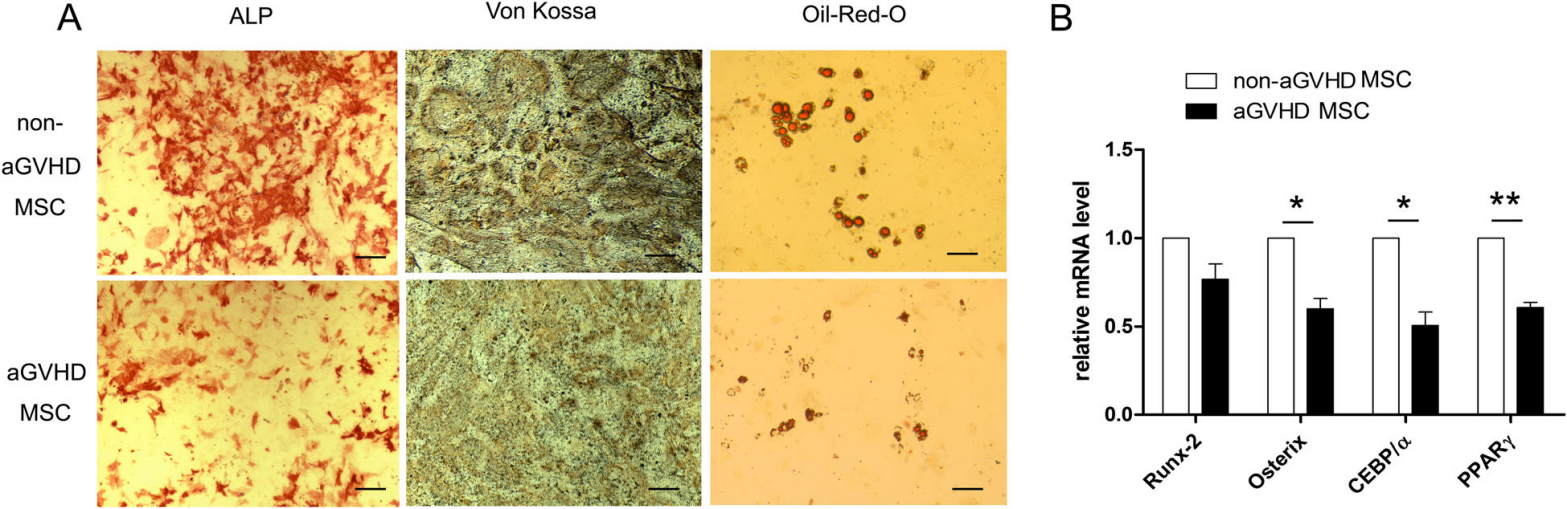
aGVHD MSCs showed impaired osteogenic and adipogenic differentiation. **a** The osteoblastogenesis and mineralization of MSCs were determined by ALP staining and von Kossa respectively. The adipogenesis of MSCs were showed by Oil-Red-O staining. The aGVHD MSCs exhibited decreased osteogenic and adipogenic activities. Bars in **a** represent 200 μm. **b** The mRNA expression of genes (Runx-2 and Osteorix) that control MSC osteogenic differentiation and genes (CEBP/α and PPARγ) that control MSC adipogenic differentiation were decreased in aGVHD MSCs relative to their counterparts in non-aGVHD MSCs. *p < 0.05, **p < 0.01, *n* = 5

As shown in Fig. 2a, the ALP activity of aGVHD MSCs remarkably decreased after culture in osteogenic medium for 2 weeks, and the number of mineralized nodules was also reduced after 4 weeks of osteogenic induction. Additionally, only a few intracellular Oil-Red-O-stained lipids accumulated after 2 weeks of adipogenic culture in aGVHD MSCs (Fig. 2a). Further, the mRNA expression levels of genes that control MSC multi-differentiation were reduced relative to their counterparts in non-aGVHD MSCs (Fig. 2b) (*p < 0.05, **p < 0.01).

### aGVHD MSCs harbored deficient hematopoietic promotion capacity

**Fig. 3:**
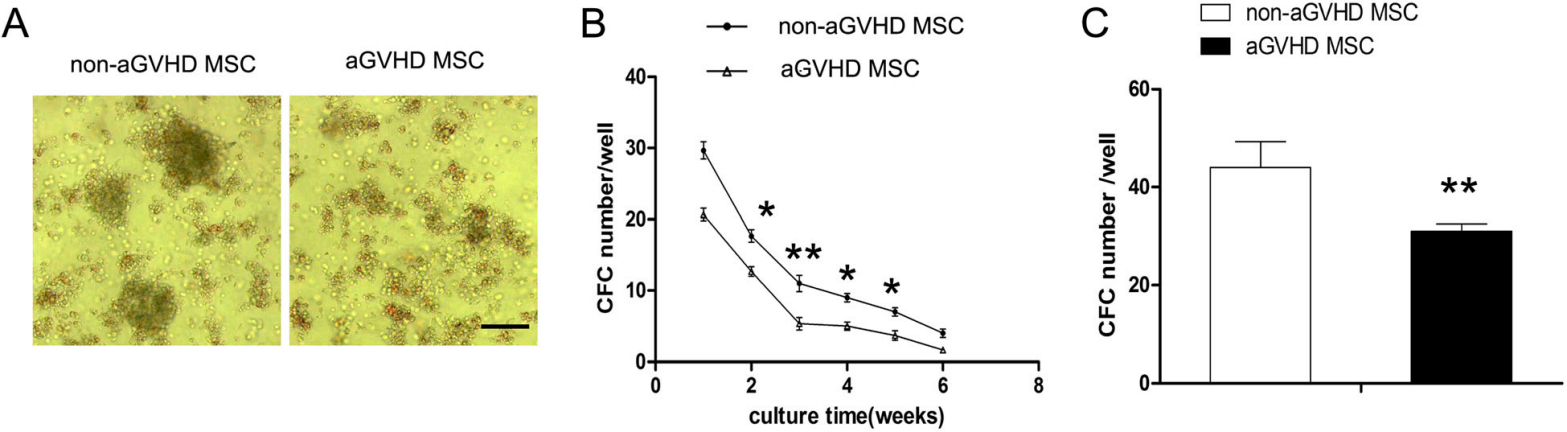
aGVHD MSCs are deficient in hematopoietic supporting capacity. **a** The morphologic characteristics of CFCs in long-term culture of hematopoietic cells were observed under an inverted light microscope. Bars in **a** represent 200 μm. **b** The CFC number in the different weeks and **c** the CFC number of the bulk culture of long-term culture-initiating cells were counted under an inverted light microscope. Less CFCs were generated in the presence of aGVHD MSCs. *p < 0.05, **p < 0.01, compared with non-aGVHD groups, *n* = 5

To investigate the effects of aGVHD on the hematopoiesis-promoting capacity of BM MSCs, an in vitro long-term hematopoietic support assay was performed. As shown in Fig. 3a, the CFCs generated by the aGVHD MSC feeders were remarkably smaller than those of non-aGVHD MSCs. In addition, the number of CFCs in the presence of aGVHD MSC feeders was significantly decreased at multiple time points (Fig. 3b). Furthermore, the results of an LTC-IC assay using bulk culture demonstrated that in the presence of aGVHD MSCs, 27.6 ± 2.6 CFCs were generated from culture, which is lower than the 42.8 ± 4.6 CFCs in non-aGVHD MSC culture (Fig. 3c) (*p < 0.05, **p < 0.01).

### TNF-α contributed to the impairment of aGVHD MSCs

**Fig. 4:**
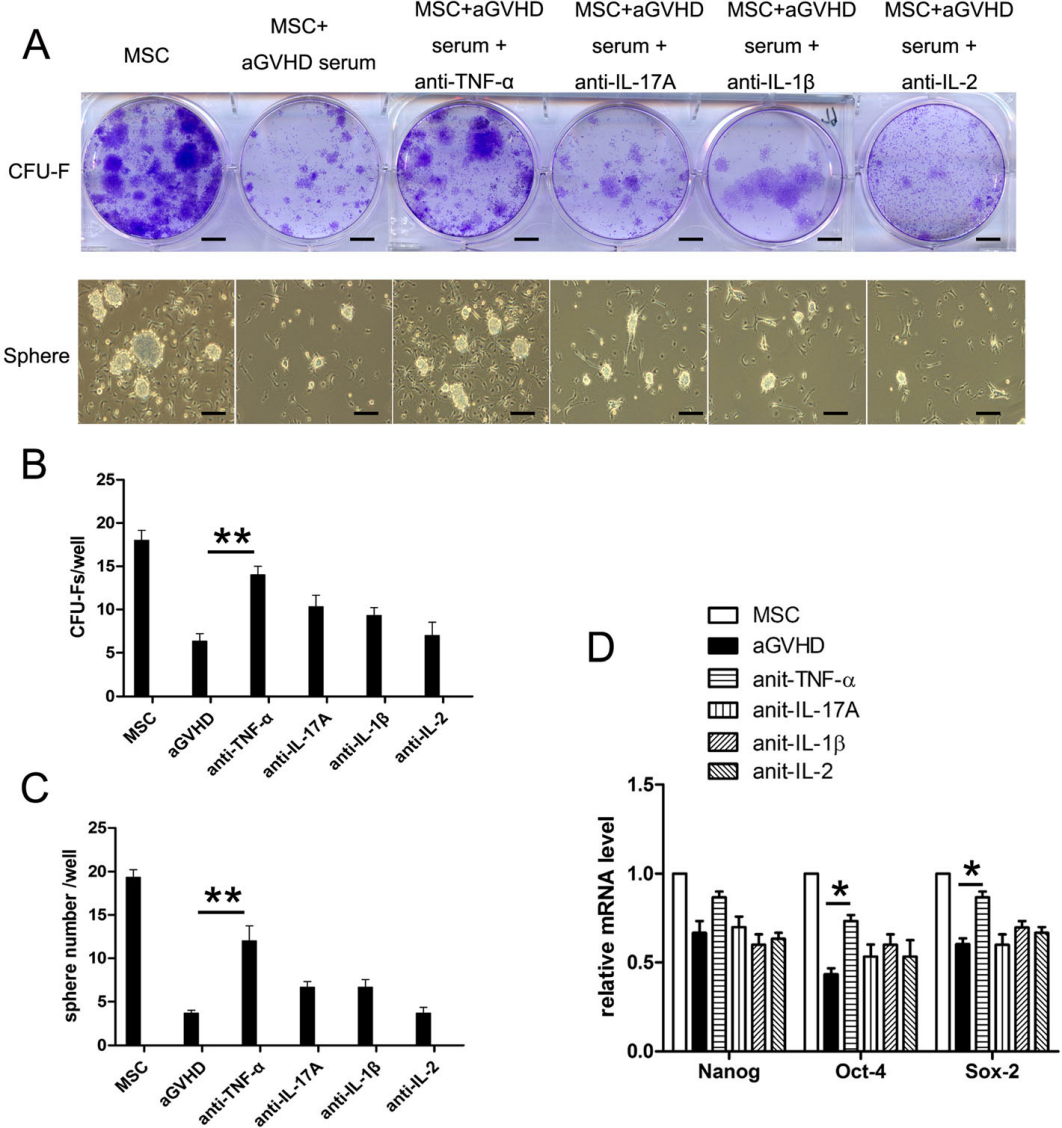
TNF-α in aGVHD BM supernatants contributed to the impairment of the self-renewal of MSCs. **a** The self-renewal activities of MSCs were assayed by CFU-F experiments and sphere formations in the presence of neutralization antibodies (anti-TNF-α, anti-IL-17A, anti-IL-1β, and anti-IL-2) and aGVHD BM supernatants. Bar in **a** upper row represents 5 mm. Bar in **a** lower row represent 200 μm. **b** The CFU-F number and **c** the cell sphere number in the TNF-α neutralization group were significantly higher than that of no antibodies group. **p < 0.01, *n* = 5. **d** The neutralization of TNF-α increased the mRNA expression of Oct-4 and Sox-2 in MSCs. **p < 0.01, compared with aGVHD groups, *n* = 5

To investigate the roles of inflammatory factors in aGVHD BM supernatants on MSC self-renewal, neutralization antibodies, including anti-TNF-α, anti-IL-17A, anti-IL-1β, and anti-2 antibodies, were used in CFU-F experiments and sphere formation. The results showed that CFU-F and sphere number were remarkably reverted in the presence of anti-TNF-α antibodies (Fig. 4a–c). In addition, the mRNA levels of Oct-4 and Sox-2 were partially rescued by TNF-α blockage (Fig. 4d) (*p < 0.05, **p < 0.01).

**Fig. 5:**
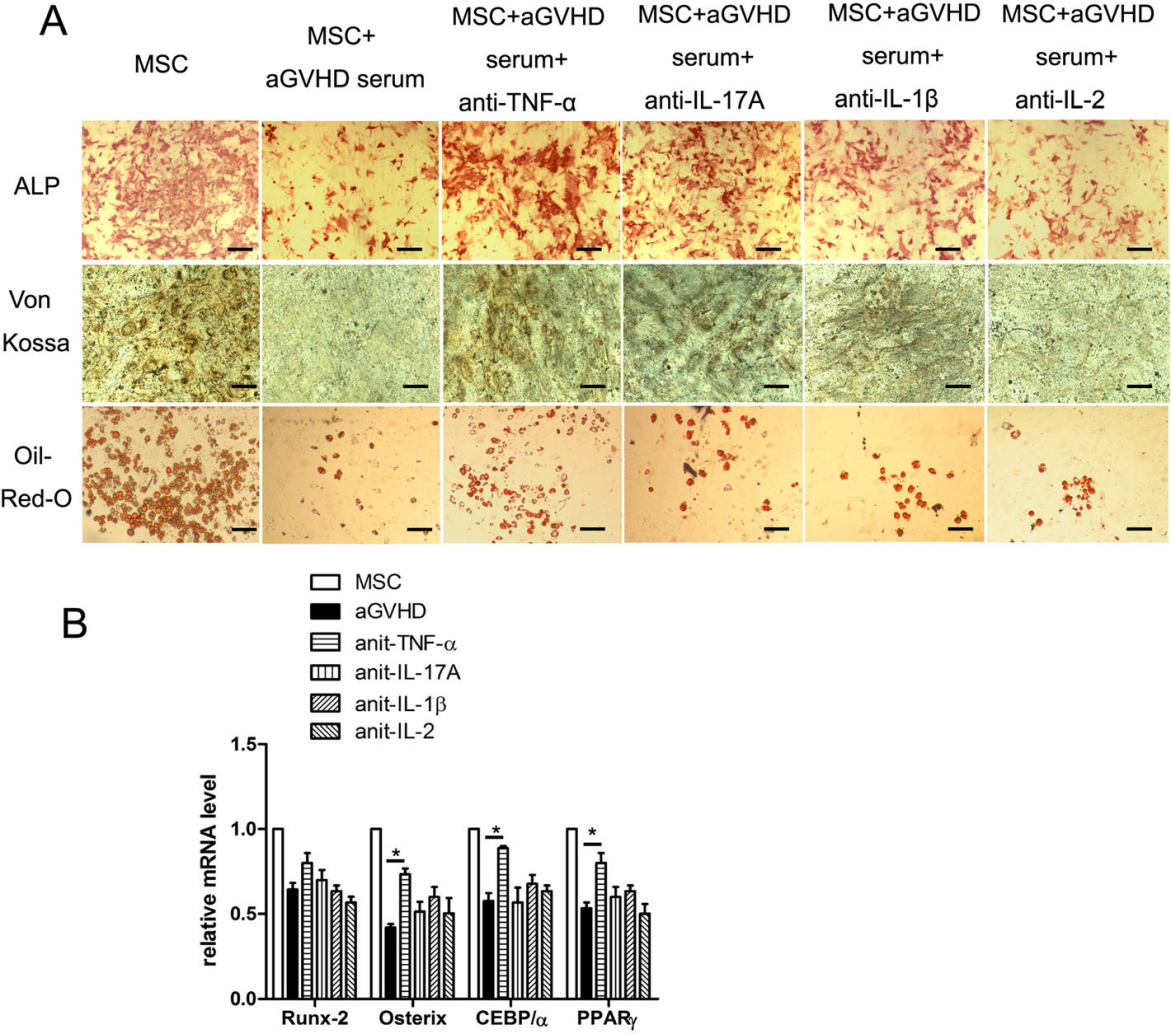
TNF-α in aGVHD BM supernatants suppressed the multi-differentiation of MSCs. **a** The addition of anti-TNF-α neutralization antibody rescued the ALP activities and mineralization as well as fat droplet development in MSCs. Bars in **a** represent 200 μm. **b** The TNF-α neutralization antibody rescued the expression of osteogenic genes Runx2 and Osteorix and the adipogenic genes CEBP/α and PPARγ. *p < 0.05, compared with aGVHD groups, *n* = 5

For a multiple differentiation assay, anti-TNF-α, anti-IL-17A, anti-IL-1β, and anti-2 antibodies were added into osteogenic and adipogenic induction cultures. The results demonstrated that TNF-α blockage increased the ALP activities and mineralization in osteogenic cultures (Fig. 5a). Additionally, the addition of an anti-TNF-α antibody increased the numbers of adipocytes in adipogenic culture. Further investigation of the expression of osteogenic and adipogenic differentiation genes validated the effects of the anti-TNF-α antibody in rescuing the multiple differentiation capacity of MSCs (Fig. 5b) (*p < 0.05).

**Fig. 6:**
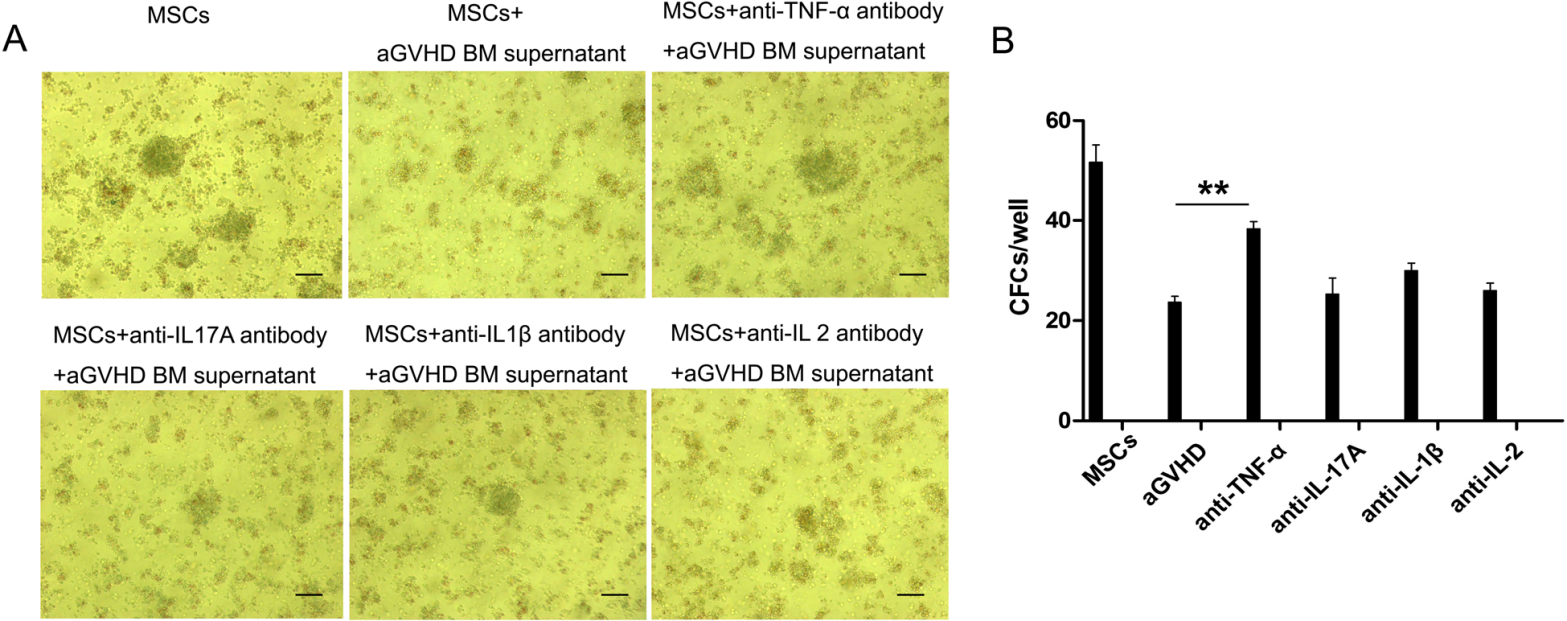
TNF-α in aGVHD BM supernatants suppressed the hematopoietic supporting capacity of MSCs. **a** The CFC morphologic characteristics in the bulk culture of long-term culture-initiating cells were observed under a reverted light microscope. Bars in **a** represent 200 μm. **b** The CFCs numbers in the presence of anti-TNF-α neutralization antibody partially recovered. **p < 0.01, compared with aGVHD groups, *n* = 5

To evaluate MSC hematopoiesis promotion capacity, an LTC-IC assay using bulk culture was performed. As showed in Fig. 6a, b, a TNF-α neutralization antibody partially rescued CFC formation (**p < 0.01).

To further determine the role of TNF-α in aGVHD bone marrow supernatants, the TNF-α concentration were assayed by ELISA. As showed in Table S3, the TNF-α levels in 15 aGVHD patients significantly higher than that of their non-aGVHD counterparts (959.2 ± 129.3 versus 56.8 ± 10.2, *p* < 0.01).

### TNF-α blockage by a recombinant human TNF-α receptor (p75)–Fc fusion protein partially rescued MSC function

**Fig. 7:**
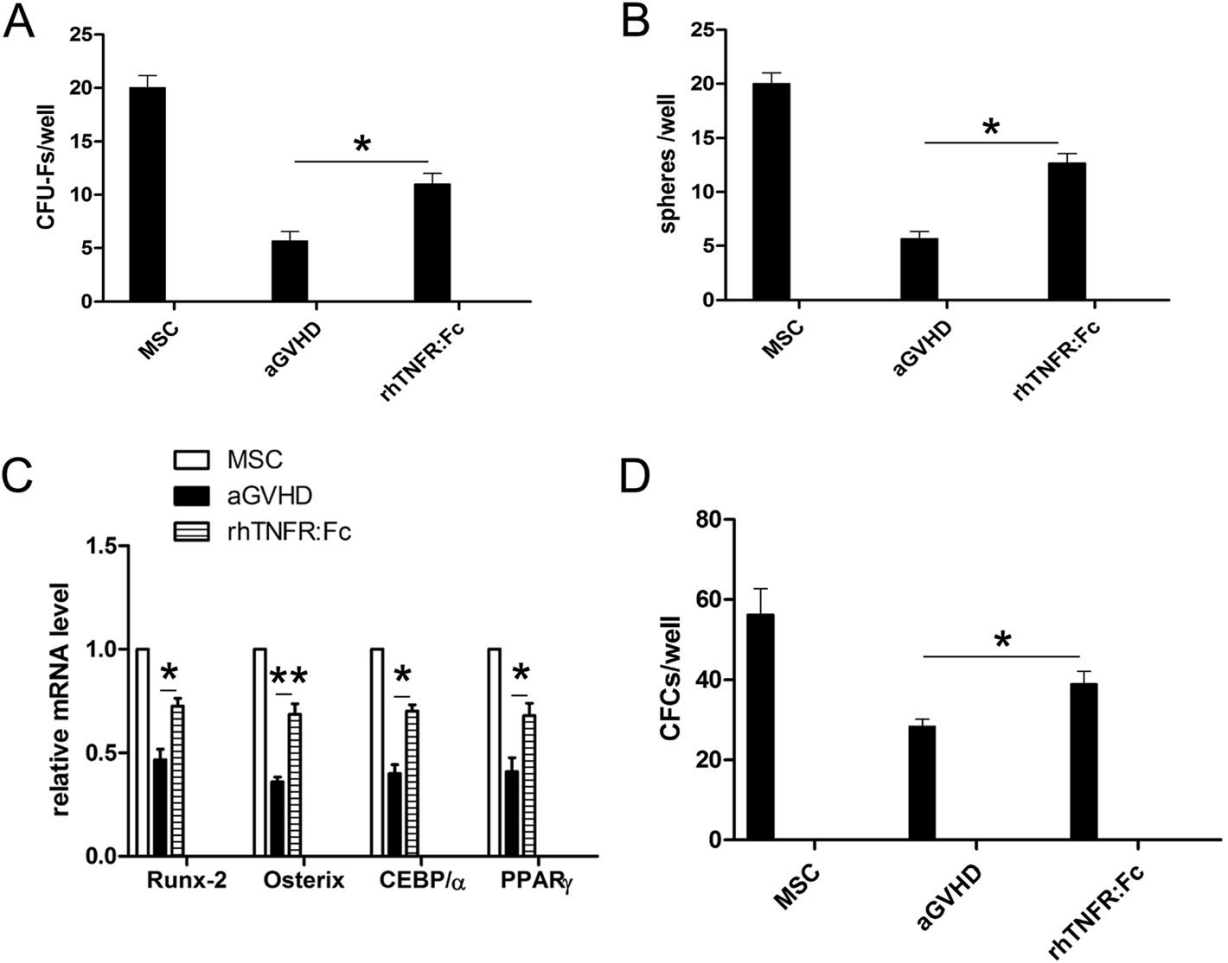
TNF-α blockage by a recombinant human TNF-α receptor (p75)–Fc fusion protein partially rescued MSC function. **a** The CFC and **b** cell sphere number significantly recovered in the presence of a recombinant human TNF-α receptor (p75)–Fc fusion protein. *p < 0.05, compared with aGVHD groups, *n* = 5. **b** The mRNA expression of Runx2, Osteorix, CEBP/α, and PPARγ were rescued by the addition of recombinant human TNF-α receptor (p75)–Fc fusion protein. *p < 0.05, **p < 0.01, compared with aGVHD groups, *n* = 5. **d** The CFC numbers in the bulk culture of long-term culture-initiating cells were partially rescued by recombinant human TNF-α receptor (p75)–Fc fusion protein. *p < 0.05, compared with aGVHD groups, *n* = 5

**Fig. 8:**
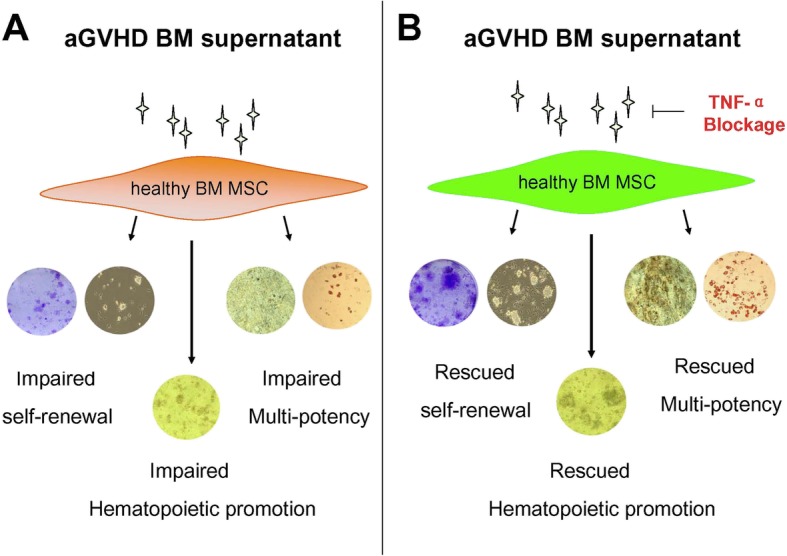
TNF-α has a pivotal role in aGVHD mediated MSC injury. **a** aGVHD induced-cytokine storm injure the self-renewal, multipotency, and hematopoietic promotion of bone marrow mesenchymal stem cells (BM MSC). **b** Blockage of tumor necrosis factor α (TNF-α) partially rescue the MSC properties

To explore whether TNF-α could be used as a novel target for protecting BM from aGVHD, a rhTNFR:Fc was added into the aGVHD BM supernatant/MSCs coculture at a concentration of 20 μg/ml or 100 μg/ml. Notably, the number of CFU-Fs and spheres remarkably recovered in a fusion protein dose-dependent manner (Fig. 7a, b). In addition, osteogenic and adipogenic differentiation partially reverted in the presence of the fusion protein (Fig. 7c). Promisingly, CFU-C formation was also rescued by the addition of the fusion protein (Fig. 7d) (*p < 0.05, **p < 0.01). Therefore, our data suggest a model of TNF-α blockage in rescue BM MSCs in aGVHD (Fig. 8).

### TNF-α changed the mRNA expression profile of human BM MSCs

Since the data above suggested that TNF-α contribute to the impairment of MSC properties in aGVHD patients, we further identified the mRNA expression profile of MSCs in the presence of TNF-α using transcriptome sequencing. 73,877 genes were examined for MSCs (*n* = 3) group and MSCs+TNF-α (*n* = 3) group. The false discovery ratio was less than 0.05, and the difference multiple greater than 2-fold change was set to define significant difference proteins. Nine hundred ten genes were upregulated and 402 genes were downregulated in the MSCs+TNF-α group compared with that of the MSCs group. The volcano plot represents the results of the differentially expressed genes. The data in Fig. S3A and B presented the differential genes in heatmap and volcano plot format between the MSCs group and the MSCs+TNF-α group.

ICAM-1, the suppressors of MSC differentiation, was significantly upregulated (4.34 ± 0.11, *p* = .000002). IL-1β, which can negatively regulate MSC differentiation and proliferation, was also increased (5.26 ± 0.15, *p* = .000001). NF-κB1 and NF-κB2, the positive regulators of NF-κB signaling, are closely related with innate immune response and cell fate and were dramatically increased (2.76 ± 0.15, *p* = .000032, for NF-κB1; 2.44 ± 0.11, *p* = .000021, for NF-κB2). BMP4, the positive regulator of bone formation, was remarkably decreased (− 2.97 ± 0.015, *p* = .0029). GDF15 encodes a secreted ligand of the transforming growth factor β (TGF-β) superfamily and binds various TGF-β receptors leading to crosstalks with BMP-signaling and was dramatically decreased (− 12.59 ± 0.11, *p* = .000001). However, the expression of numerous hematopoietic factors including IL-6, IL-8, colony-stimulating factor 1, IL-34, IL-11, IL-15, and IL-23A significantly increased, which seemly is not consistent with the cellular phenomenon. We assume that not only the expression level of hematopoietic factors but also an appropriate cytokine profile is important to maintain normal hematopoiesis. The results of Q-PCR validated the significant gene (ICAM-1, IL-1β, BMP4, and IL-6) expression of RNA sequencing (Fig. S4A and B) (**p < 0.01).

Using Gene Ontology (GO) database, genes can be classified into gene functional enrichment and analysis according to their biological processes. All differentially abundant genes between the MSCs and MSCs+TNF-α were mainly concerned with lymphocyte differentiation, osteoclast differentiation, cell adhesion, cytokine-cytokine receptor interaction, etc. (Fig. S4A and B). Further analysis suggested that TNF-α signaling pathway, Toll-like receptor signaling pathway, and cytokine-cytokine receptor signaling pathway were closely involved in the TNF-α-induced change of MSC properties.

## Discussion

In the current study, we demonstrated the suppressive effects of aGVHD on the stemness and hematopoiesis promotion capacity of recipient BM MSCs. Additionally, we found that TNF-α blockage was capable of rescuing aGVHD-induced endogenous MSC injuries.

Although BM is known to be a target organ post-allogeneic HSCT, the underlying mechanisms remain unclear. For a long time, most of the experimental and clinical studies mainly focused on the differentiated cellular components, including osteoblasts and endothelial cells, in the BM compartments until Copland et al. reported that BM MSCs of aGVHD and chronic GVHD patients exhibited a normal phenotype, differentiation potential, and immune-suppressive properties [18]. The report provided important information that functional MSCs remained present in the BM niche of GVHD patients. However, the BMs were harvested from GVHD patients and healthy volunteers instead of non-aGVHD patients who received allo-HSCT. In addition, the self-renewal of MSCs in GVHD patients was not reported in this study. Furthermore, the authors stated that recipient-derived MSCs from GVHD patients may represent a viable option for clinical testing but did not assess the role of MSCs in BM niche reconstitution post-HSCT. At nearly the same time, Wang et al. demonstrated that MSCs from patients with chronic GVHD displayed normal phenotype and function relative to their counterparts from healthy donors, but their reduced frequency in the BM mononuclear cell fraction was observed in these patients [19]. Another subsequent study reported that the number of Nestin-positive stem cells in BM biopsies was significantly reduced in aGVHD patients compared with patients who did not have aGVHD. Notably, the density of Nestin-positive stem cells returned to normal after aGVHD resolved. Therefore, pioneering studies strongly indicate an impairment of BM MSCs in aGVHD patients, but further functional changes of MSCs and, most importantly, the mechanisms and rescue strategies remain unknown.

Based on the parallel analysis of BM MSCs from aGVHD patients and non-aGVHD patients post-allo-HSCT, we first evaluated MSC stemness, which controls the MSC pool and cell function, to determine the reconstitution of the stromal BM niches. In the present study, we found that aGVHD MSCs exhibited lagged cell proliferation relative to non-aGVHD MSCs, which is comparable to the data of Copland et al. We used BM mononuclear cells instead of MSCs to develop primary cell colonies, and it is possible that there is a different frequency of MSCs instead of an intrinsic alteration of self-renewal of aGVHD MSCs. To exclude the ambiguous results, the self-renewal of bone marrow MSCs was assayed by both CFU-F and sphere formation experiments in MSC culture medium so as to exclude the possibility of other progenitor-derived cell colonies. In addition, we performed the primary and secondary colony formation experiments to evaluate the cell self-renewal of MSCs. The results showed that the number of CFU-Fs and spheres reduced in the aGVHD groups, which indicated decreased self-renewal of aGVHD MSCs. The analysis of the expression of Nanog, Oct-4, and Sox-2 further confirmed the MSC reduction in aGVHD patients. These data are controversial relative to the reports of Copland et al. and Wang et al., partially because the BM samples were from chronic GVHD and unparalleled HSCT patients, which may lead to the different findings. Notably, our data are comparable to BM biopsy-based results showing that the number of Nestin-positive stem cells decreased in aGVHD patients [18–20]. Thus, our data suggested that aGVHD MSCs exhibited an impaired self-renewal capacity.

In addition to self-renewal capacity, multipotency is another important feature of MSC stemness. As one of the seed sources of osteoblasts for endosteal niches, osteogenically differentiated MSCs are indispensable for BM niche maintenance and reconstitution [21, 22]. In addition, adipocytes are regarded as key keepers of quiescent HSCs in the BM niche [23, 24]. Therefore, we investigated whether aGVHD affects the osteogenic and adipogenic differentiation of MSCs. We found that aGVHD MSCs exhibited decreased multipotency, which may hamper the reconstitution of the stromal BM niche and is helpful to understand the delay of hematopoiesis recovery of aGVHD patients.

Current knowledge regarding hematopoietic supportive capacity of BM MSCs in aGVHD was mostly assayed by experimental animals. On the contrary, there is little information available about the role of MSCs in the hematopoiesis recovery of aGVHD patients. In the current study, we directly evaluated the hematopoiesis promotion capacity of aGVHD MSCs. Both the weekly CFC assay and the LTC-IC assay using bulk culture were carried out. The results proved a remarkable decline of hematopoiesis promotion of aGVHD MSCs. Thus, we reported for first time that aGVHD MSCs exhibited deceased hematopoiesis promotion capacity, which may delay the rebuilding of BM niche function and contribute to aGVHD-induced BM suppression.

Although we found that aGVHD damaged MSC stemness and hematopoiesis promotion capacity, the underlying mechanisms remained to be elucidated. Increasing amounts of data have demonstrated that aGVHD-induced cytokine storm in the blood is responsible for target organ damage [6, 8, 25]. Therefore, in the present study, BM supernatants were used to treat MSCs to investigate the potential mechanisms of aGVHD on MSCs. TNF-α is one of the inflammatory factors of cytokine storm and has been demonstrated to be closely involved in the pathological process of aGVHD. Huang et al. reported that serum TNF-α levels were significantly higher in patients with grades II–IV aGVHD than in those with grade 0 or I aGVHD and suggested that TNF-α may be useful for the diagnosis of aGVHD [26]. In addition, accumulated evidence has demonstrated that TNF-α plays a pivotal role in the amplification of tissue injury during GVHD, but the role of TNF-α in GVHD-associated myelosuppression remains to be elucidated [27–29]. By using neutralization antibodies towards inflammatory factors, including TNF-α, IL-17A, IL-2, and IL-1β, we identified TNF-α as a key factor that contributed to the impairment of MSC stemness. In addition, our results further showed that TNF-α damaged the hematopoiesis promotion capacity of MSCs. These data constitute the first report that aGVHD BM microenvironments cause endogenous MSC injury and suggest that it is possible to rescue BM aGVHD by targeting TNF-α. Our data are different from a previous study that incubated MSCs in mixed lymphocyte reaction (MLR) supernatants including inflammatory factors [30]. Briefly, Fasslrinner et al. found that the proliferative activities of MLR-treated MSCs increased, but cell differentiation shifted towards the osteogenic lineage [30]. We assume that the differences in data may be caused by the supernatants used for MSC culture. In the present study, we collected BM supernatants from aGVHD patients, which may contain higher concentrations of inflammatory factors than those of in vitro MLR supernatants. Most importantly, aGVHD patient-derived samples may include more categories of cytokines that from effector cells and damaged tissues. Interestingly, we found that aGVHD BM supernatants partially injured the hematopoiesis promotion capacity of TNF-α. The result is consistent with a report of Fasslrinner et al. [30], showing that the ability of MLR-treated MSCs to support HSCs in vitro was reduced. Thus, the underlying mechanisms that regulate MSC hematopoiesis-supporting capacity need further investigation for clarification.

In the past two decades, blockage of TNF-α has been clinically applied in the treatment of inflammatory diseases, including rheumatoid arthritis, multiple myeloma, and psoriatic arthritis [31–33], and remarkable therapeutic effects have been observed. However, the importance of TNF-α in GVHD-associated myelosuppression has not yet been validated in detail. Therapeutic strategies targeting TNF-α include agents modulating TNF-α signaling, anti-TNF-α monoclonal antibodies, and soluble TNF receptors. TNF has at least two distinct receptors (a 55-kDa (p55) and a 75-kDa (p75) protein) on cell surfaces and in soluble forms, which serve as physiologic regulators of the inflammatory response. Based on our original findings that TNF-α in the BM supernatant damages the stemness and hematopoiesis promotion capacity of MSCs and according to previously published results showing that TNF-α in MLR supernatant affects the multiple differentiation and hematopoietic supporting capacity of MSCs [30], we added clinically used recombinant human TNF receptor (p75)–Fc fusion protein as decoy receptors to block TNF signaling and to protect MSC properties. Notably, TNF-α blockage significantly improved the impaired MSC properties, which strongly suggested that this may be a novel strategy to promote the BM microenvironment and hematopoiesis reconstitution by targeting TNF-α.

Though the data above have demonstrated the pivotal role of TNF-α in aGVHD-mediated BM damage, the molecular mechanisms underlying TNF-α control of the stemness and hematopoiesis-promoting capacity of MSCs need to be further clarified. In the current study, the comprehensive results of a high-throughput RNA sequencing analysis suggested that numerous genes including ICAM-1, IL-1β, BMP4, and GDF15 may be responsible for the special mechanisms of TNF-α mediated effects of stem cell properties [34–38]. The further GO annotation revealed the biological process of the differential abundant genes. However, the MSCs for high-throughput RNA sequencing analysis were pretreated by a higher concentration of TNF-α, and the concentration is high and is likely different to that in aGVHD patients. In addition, the BM supernatants were permanently added into the MSCs culture system instead of pretreatment in the current study, which may result in different biological effects. Thus, we are aware that the results of RNA sequencing could not fully explain the complete mechanisms of the impairments on MSC properties and more functional experiments are needed for further clarifications.

Nevertheless, we must acknowledge that there are several limitations of our study. First, our results are based on in vitro experiments, and in vivo studies will be helpful to validate our findings. Second, the molecular mechanisms underlying TNF-α control of the stemness and hematopoiesis promotion of MSCs should be clarified in further investigations, and more functional explorations may be helpful to address this issue. Third, additional studies are needed to determine whether other factors contribute to aGVHD-mediated suppression of MSC properties in the future. Fourth, the number of aGVHD samples used in the current study should be enlarged to reinforce the findings. Notably, further controls such as aGVHD MSCs cultured in BM supernatants from healthy BM so as to assess the reversibility of the aGVHD MSCs should be included in further studies.

## Conclusion

In the present study, we demonstrate that the endogenous BM MSCs of recipients are cellular targets of human aGVHD. In addition, our study reveals a potential role of TNF-α in MSC injury and indicates that blockage TNF-α may be helpful to maintain bone marrow niche of aGVHD patients. Importantly, a comprehensive analysis of the BM MSC properties in human aGVHD is needed to further investigate and clarify these results.

## Supplementary information

**Additional file 1: Figure S1.** aGVHD MSCs share similar morphological and immunophenotypic characteristics with non-aGVHD MSCs. (A) The morphological characteristics were observed under an inverted light microscope. The fibroblast-like MSCs appeared at day 7 and the cell colonies were remarkable at about day 14. Bars in Fig. S1A represent 200 μm. (B) The representative results of flow cytometry showed that both aGVHD MSCs and non-aGVHD MSCs highly expressed the stromal marker (CD44) and stem cell markers (CD73, CD105, CD166) but lowly expressed the endothelial marker (CD31), the hematopoietic marker (CD45), *n* = 5.

**Additional file 2: Figure S2.** aGVHD MSCs exhibited decreased cell proliferation. (A) Trypan blue exclusion cell counting and (B) a CCK-8-based cell proliferation assay were performed. aGVHD MSCs exhibited decreased proliferative capacity relative to that of non-aGVHD MSCs .*, *p* < 0.05, **, *p* < 0.01, n = 5.

**Additional file 3: Figure S3.** The profile of gene change in human BM MSCs in the presence of TNF-α. (A) The heatmap and (B) the volcano plot showed TNF-α induced comprehensive gene expression change in human BM MSCs. compared with no TNF-α groups, *n* = 3.

**Additional file 4: Figure S4.** TNF-α changed the mRNA expression profile of human BM MSCs. (A) The expression of pathway in human BM MSCs after primed by TNF-α(n = 3). (B) The gene changes were validated by Q-PCR. **, p < 0.01, compared with no TNF-α groups, n = 5.

**Additional file 5: Table S1**. Patient Characteristics.

**Additional file 6: Table S2.** Primer sequences.

**Additional file 7: Table S3.** Serum TNF-α levels in patients with grade II–IV aGVHD after bone marrow transplant.

## Data Availability

The datasets used and/or analyzed during the current study are available from the corresponding author on reasonable request.

## Abbreviations

aGVHD: Acute graft versus host disease
BM: Bone marrow
CFC: Colony-forming cell
CFU-F assay: Colony-forming unit fibroblast formation assay
FBS: Fetal bovine serum
FTIC: Fluorescein isothiocyanate
G-CSF: Granulocyte colony-stimulating factor
HSCT: Hematopoietic stem cell transplantation
IFN-γ: Interferon γ
LTC-ICs: Long-term culture-initiating cells
MHC: Major histocompatibility complex
MSCs: Mesenchymal stem cell
PBSCs: Peripheral blood stem cells
PCR: Polymerase chain reaction
PE: Phycoerythrin
rhTNFR:Fc: Human tumor necrosis factor (TNF-α) receptor-Ig fusion protein
TNF-α: Tumor necrosis factor α
