# Supplementary figures and images for: Tumor necrosis factor α in aGVHD patients contributed to the impairment of recipient bone marrow MSC stemness and deficiency of their hematopoiesis-promotion capacity
Source: Stem Cell Res Ther. 2020 Mar 17;11:119. doi: 10.1186/s13287-020-01615-9 (PMC7079531; doi:10.1186/s13287-020-01615-9)

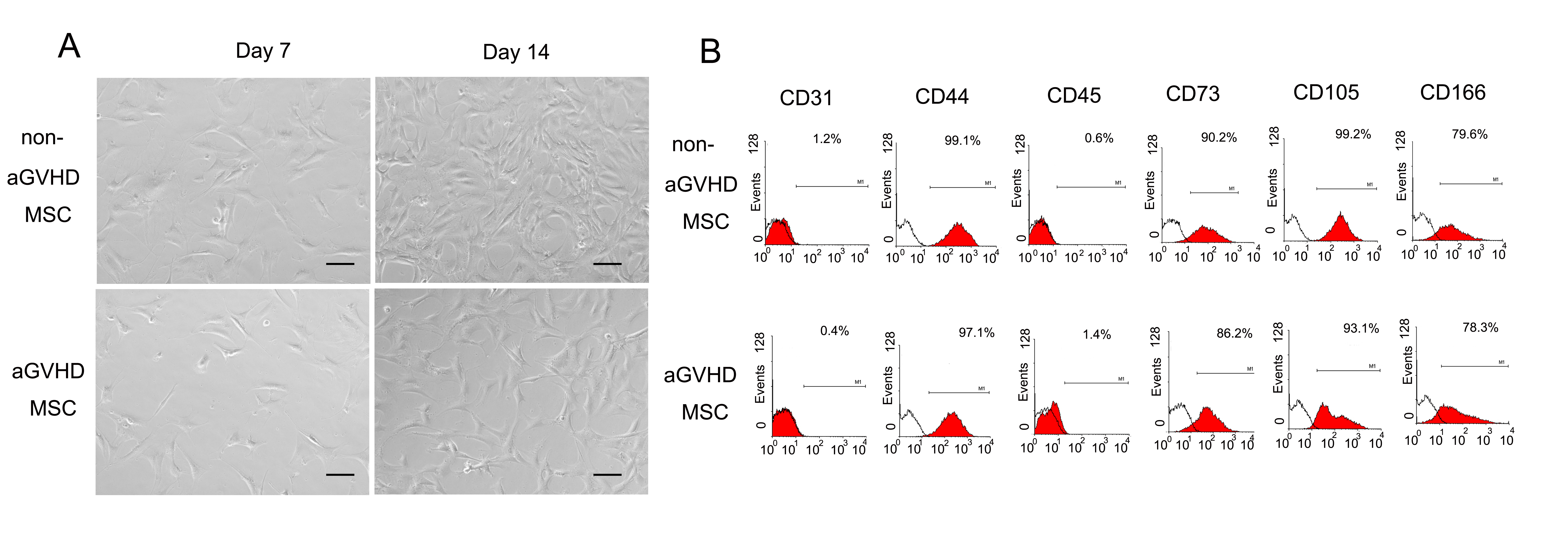

Supplement: Supplementary file 1 — Additional file 1: Figure S1. aGVHD MSCs share similar morphological and immunophenotypic characteristics with non-aGVHD MSCs. (A) The morphological characteristics were observed under an inverted light microscope. The fibroblast-like MSCs appeared at day 7 and the cell colonies were remarkable at about day 14. Bars in Fig. S1A represent 200 μm. (B) The representative results of flow cytometry showed that both aGVHD MSCs and non-aGVHD MSCs highly expressed the stromal marker (CD44) and stem cell markers (CD73, CD105, CD166) but lowly expressed the endothelial marker (CD31), the hematopoietic marker (CD45), n = 5. [file 13287_2020_1615_MOESM1_ESM.jpg]

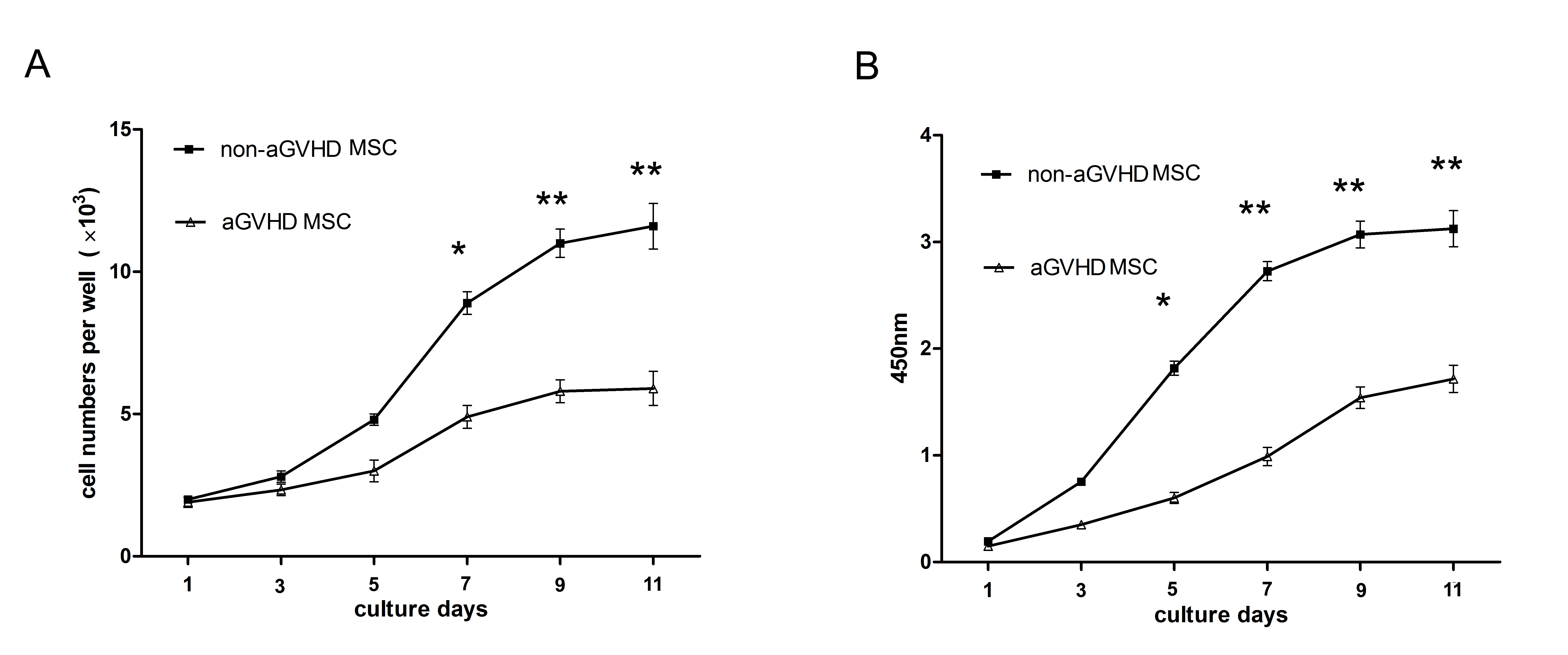

Supplement: Supplementary file 2 — Additional file 2: Figure S2. aGVHD MSCs exhibited decreased cell proliferation. (A) Trypan blue exclusion cell counting and (B) a CCK-8-based cell proliferation assay were performed. aGVHD MSCs exhibited decreased proliferative capacity relative to that of non-aGVHD MSCs .*, p < 0.05, **, p < 0.01, n = 5. [file 13287_2020_1615_MOESM2_ESM.jpg]

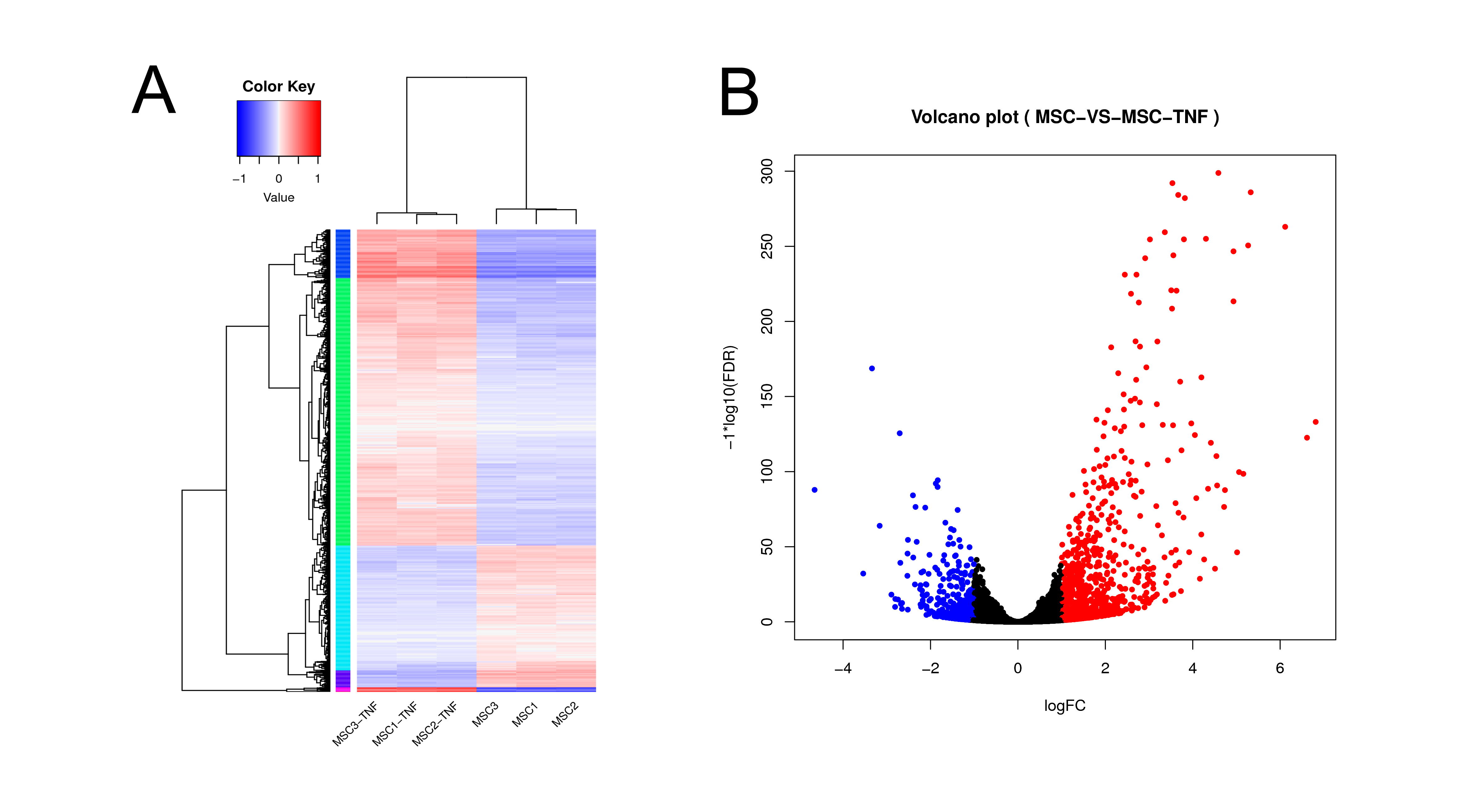

Supplement: Supplementary file 3 — Additional file 3: Figure S3. The profile of gene change in human BM MSCs in the presence of TNF-α. (A) The heatmap and (B) the volcano plot showed TNF-α induced comprehensive gene expression change in human BM MSCs. compared with no TNF-α groups, n = 3. [file 13287_2020_1615_MOESM3_ESM.jpg]

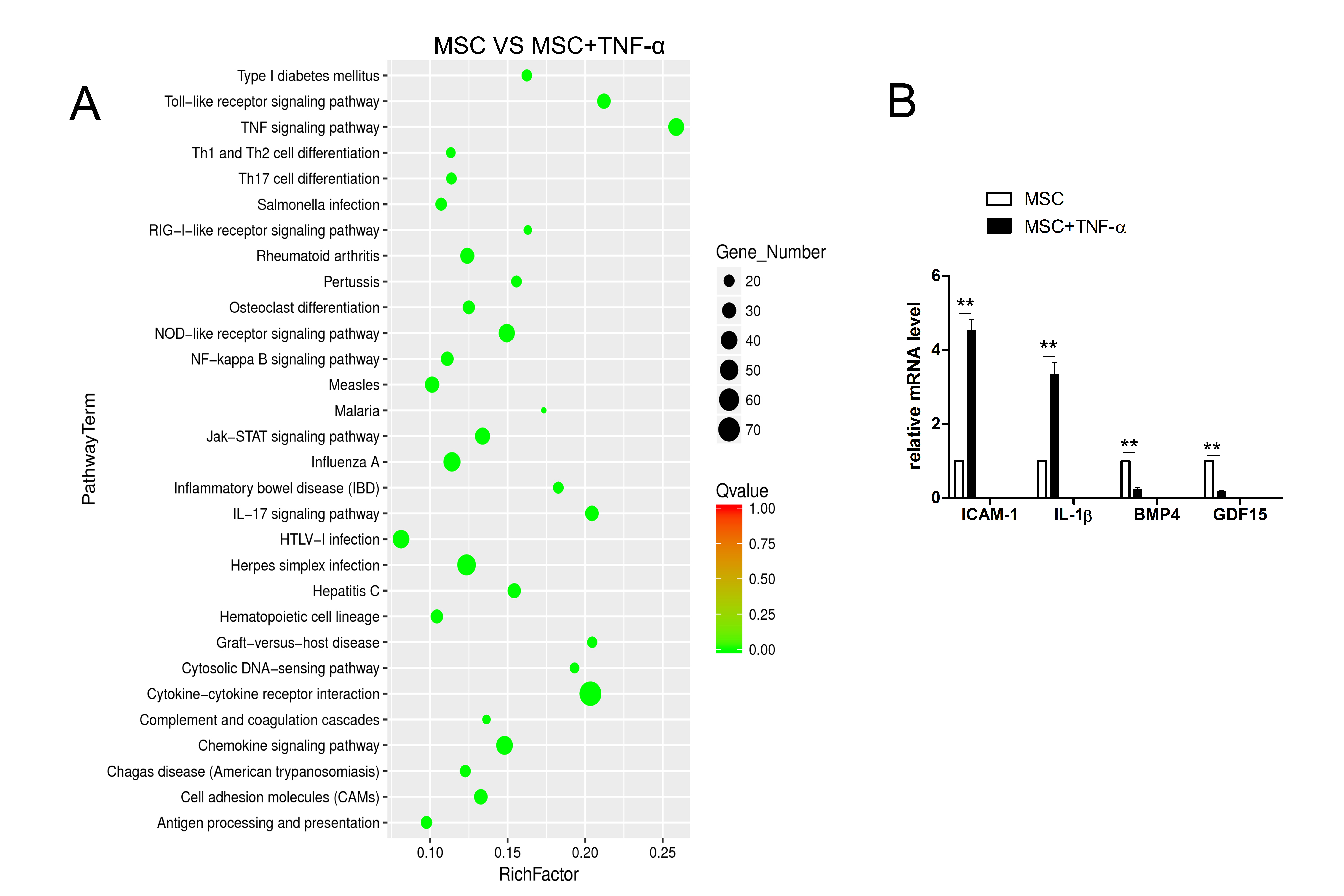

Supplement: Supplementary file 4 — Additional file 4: Figure S4. TNF-α changed the mRNA expression profile of human BM MSCs. (A) The expression of pathway in human BM MSCs after primed by TNF-α(n = 3). (B) The gene changes were validated by Q-PCR. **, p < 0.01, compared with no TNF-α groups, n = 5. [file 13287_2020_1615_MOESM4_ESM.jpg]
