# Supplementary material for: Tumor necrosis factor α in aGVHD patients contributed to the impairment of recipient bone marrow MSC stemness and deficiency of their hematopoiesis-promotion capacity
Source: Stem Cell Res Ther. 2020 Mar 17;11:119. doi: 10.1186/s13287-020-01615-9 (PMC7079531; doi:10.1186/s13287-020-01615-9)
Supplement: Supplementary file 5 — Additional file 5: Table S1. Patient Characteristics. [file 13287_2020_1615_MOESM5_ESM.docx]

**Table S1: Patient Characteristics**

| Patient number | Age  (yr) | Gender  recipient/  donor | Disease | a GVHD( II to IV ) | HLA compatibili-ty | Preparatory  regimen | Transplant  source | | GvHD  prophylaxis |
| --- | --- | --- | --- | --- | --- | --- | --- | --- | --- |
| 1 | 10-20 | M/Mother | ALL | N | 3/6 | BU+CY+Ara-C | | PB+BM | CAMM |
| 2 | 20-30 | M/Father | CML  -AP | N | 3/6 | BU+CY+Ara-C | | PB+BM | CAMM |
| 3 | 0-10 | M/Mother | AML | N | 3/6 | BU+CY+Ara-C | | PB+BM | CAMM |
| 4 | 0-10 | M/Mother | ALL | N | 3/6 | BU+CY+Ara-C | | PB+BM | CAMM |
| 5 | 40-50 | M/Sister | ALL | N | 3/6 | BU+CY+Ara-C | | PB+BM | CAMM |
| 6 | 0-10 | M/Father | ALL | N | 3/6 | BU+CY+Ara-C | | PB+BM | CAMM |
| 7 | 0-10 | F/Father | ALL | N | 3/6 | BU+CY+Ara-C | | PB+BM | CAMM |
| 8 | 10-20 | F/Mother | AML | N | 3/6 | BU+CY+Ara-C | | PB+BM | CAMM |
| 9 | 10-20 | M/Mother | CML | N | 3/6 | BU+CY+Ara-C | | PB+BM | CAMM |
| 10 | 30-40 | F/Son | AML | N | 3/6 | BU+CY+Ara-C | | PB+BM | CAMM |
| 11 | 40-50 | M/Sister | ALL | N | 4/6 | BU+CY+Ara-C | | PB+BM | CAMM |
| 12 | 20-30 | M/Brother | CML  -AP | N | 6/6 | BU+ Y | | PB | CsA, MTX |
| 13 | 20-30 | M/Brother | ALL | N | 6/6 | BU+CY | | PB | CsA, MTX |
| 14 | 0-10 | M/ Unrelated  donor | ALL | N | 6/6 | BU+CY+Ara-C | | PB | CAMM |
| 15 | 10-20 | M/Brother | AML | N | 6/6 | BU+CY | | PB | CsA, MTX |
| 16 | 10-20 | M/Mother | AML | Y | 4/6 | BU+CY+Ara-C | | PB+BM | CAMM |
| 17 | 20-30 | M/Father | CML  -CP | Y | 3/6 | BU+CY+Ara-C | | PB+BM | CAMM |
| 18 | 30-40 | M/Brother | CMML | Y | 3/6 | BU+CY+Ara-C | | PB+BM | CAMM |
| 19 | 30-40 | M/Father | AML | Y | 3/6 | BU+CY+Ara-C | | PB+BM | CAMM |
| 20 | 10-20 | M/Father | ALL | Y | 3/6 | BU+CY+Ara-C | | PB+BM | CAMM |
| 21 | 10-20 | M/Father | ALL | Y | 3/6 | BU+CY+Ara-C | | PB+BM | CAMM |
| 22 | 10-20 | M/Father | ALL | Y | 3/6 | BU+CY+Ara-C | | PB+BM | CAMM |
| 23 | 10-20 | M/Father | AML | Y | 4/6 | BU+CY+Ara-C | | PB+BM | CAMM |
| 24 | 30-40 | F/Sister | ALL | Y | 3/6 | BU+CY+Ara-C | | PB+BM | CAMM |
| 25 | 0-10 | F/Mother | ALL | Y | 3/6 | BU+CY+Ara-C | | PB+BM | CAMM |
| 26 | 10-20 | F/Mother | ALL | Y | 3/6 | BU+CY+Ara-C | | PB+BM | CAMM |
| 27 | 10-20 | M/Mother | ALL | Y | 3/6 | BU+CY+Ara-C | | PB+BM | CAMM |
| 28 | 40-50 | M/Sister | ALL | Y | 4/6 | BU+CY+Ara-C | | PB+BM | CAMM |
| 29 | 10-20 | M/Father | ALL | Y | 3/6 | BU+CY+Ara-C | | PB+BM | CAMM |
| 30 | 20-30 | M/Father | ALL | Y | 4/6 | BU+CY+Ara-C | | PB+BM | CAMM |

F, female; M, male; AML, acute myelogenous leukemia; ALL, acute lymphocytic leukemia; CML, chronic myelogenous leukemia; AP: accelerating phase; Bu, Busulfan (12 mg/kg for haplo-HSCT; 16 mg/kg for HLA –identical HSCT); CTX, cyclophosphamide (120mg/kg); Ara-C, (8g/m^2^); BM, bone marrow; PB, peripheral blood; CAMM, cyclosporine A + antithymocyte globulin + methotrexate + mycopheno1ate mofetil; GVHD, graft-versus-host disease.
