# Supplementary material for: Tumor necrosis factor α in aGVHD patients contributed to the impairment of recipient bone marrow MSC stemness and deficiency of their hematopoiesis-promotion capacity
Source: Stem Cell Res Ther. 2020 Mar 17;11:119. doi: 10.1186/s13287-020-01615-9 (PMC7079531; doi:10.1186/s13287-020-01615-9)
Supplement: Supplementary file 6 — Additional file 6: Table S2. Primer sequences. [file 13287_2020_1615_MOESM6_ESM.docx]

| genes | primer sequences | Annealing temperature |
| --- | --- | --- |
| GADPH  Nanog  Oct4  Sox2  Runx2  Osterix  CEBP/α  PPARγ  ICAM-1  IL-1β  BMP4  GDF15 | forward, 5′- CCAAGGAGTAAGACCCCTGG -3′  reverse, 5′- TGGTTGAGCACAGGGTACTT-3′  forward, 5′- TGAGTGTGGATCCAGCTTGT-3′  reverse, 5′- TCTCTGCAGAAGTGGGTTGT-3′  forward, 5′- GGTCCGAGTGTGGTTCTGTA-3′  reverse, 5′- CGAGGAGTACAGTGCAGTGA-3′  forward, 5′- CATGTCCCAGCACTACCAGA-3′  reverse, 5′- TACCGGGTTTTCTCCATGCT-3′  forward, 5′- CTGTGGTTACTGTCATGGCG-3′  reverse, 5′- AGGTAGCTACTTGGGGAGGA-3′  forward, 5′- CCCTCCCTTTTCCCACTCAT-3′  reverse, 5′- CATGGATGCCTGCCTTGTAC-3′  forward, 5′- GGAGGGTCTCTAGTTCCACG-3′  reverse, 5′- CCCACAGCCAGATCTCTAGG-3′  forward, 5′- TTGCAGTGGGGATGTCTCAT-3′  reverse, 5′- TTTCCTGTCAAGATCGCCCT-3′  forward, 5′- AAGATCAAATGGGGCTGGGA-3′  reverse, 5′- AATGTATGTGGGTGGGGAGG-3′  forward, 5′- GGAGAATGACCTGAGCACCT-3′  reverse, 5′- GGAGGTGGAGAGCTTTCAGT-3′  forward, 5′- GCCAGCATGTCAGGATTAGC-3′  reverse, 5′- AATCCAGTCATTCCAGCCCA-3′  forward, 5′- CAGCTACAATCCCATGGTGC-3′  reverse, 5′- TCAGGAACCTTGAGCCCATT-3′ | 60 ℃ |

**Table S2: Primer sequences**
