# Supplementary material for: Tumor necrosis factor α in aGVHD patients contributed to the impairment of recipient bone marrow MSC stemness and deficiency of their hematopoiesis-promotion capacity
Source: Stem Cell Res Ther. 2020 Mar 17;11:119. doi: 10.1186/s13287-020-01615-9 (PMC7079531; doi:10.1186/s13287-020-01615-9)
Supplement: Supplementary file 7 — Additional file 7: Table S3. Serum TNF-α levels in patients with grade II–IV aGVHD after bone marrow transplant. [file 13287_2020_1615_MOESM7_ESM.docx]

**Table S3: Serum TNF-α levels in patients with grade II–IV aGVHD after bone marrow transplant.**

| Non aGVHD | n | TNF-α (pg/ml) | aGVHD( II to IV ) | n | TNF-α (pg/ml) |
| --- | --- | --- | --- | --- | --- |
|  | 15 | 56.8±10.2 |  | 15 | 959.2±129.3 |

GVHD, graft-versus-host disease, TNF-α, tumor necrosis factor α.
